# Kinetic Alfvén waves and auroral particle acceleration: a review

**DOI:** 10.1007/s41614-022-00111-2

**Published:** 2023-01-07

**Authors:** R. L. Lysak

**Affiliations:** School of Physics and Astronomy, Minnesota Institute for Astrophysics, University of Minnesota, Minneapolis, MN USA

**Keywords:** Kinetic Alfvén waves, Auroral particle acceleration, Magnetohydrodynamics, Planetary magnetospheres

## Abstract

Shear mode Alfvén waves are the carriers of field-aligned currents in the auroral zones of Earth and other planets. These waves travel along the magnetic field lines, coupling the outer magnetosphere with the ionosphere. However, in ideal magnetohydrodynamic (MHD) theory, the shear mode Alfvén wave does not carry a parallel electric field that could accelerate auroral particles. This can be modified by including kinetic effects, which lead to a parallel electric field when the perpendicular wavelength becomes comparable to the electron inertial length or the ion acoustic gyroradius. These small perpendicular wavelengths can be formed by phase mixing, ionospheric feedback, or nonlinear effects. Kinetic Alfvén waves are further constrained by their interaction with the ionosphere, which acts as a reflector for these waves. In addition, the strong plasma gradients in the topside ionosphere form an effective resonator that leads to fluctuations on time scales of seconds. These rapidly changing parallel electric fields can lead to broadband acceleration of auroral electrons, often called the Alfvénic aurora. Such interactions do not only take place in Earth’s magnetosphere, but have also been observed in Jupiter’s magnetosphere by the Juno satellite.

## Introduction

The shear mode Alfvén wave, first identified by Alfvén (1942), is a wave mode of ideal magnetohydrodynamics (MHD). These waves propagate along magnetic field lines, making them an ideal conduit for the electrodynamic coupling of the magnetosphere and ionosphere. Alfvén waves carry a net Poynting flux toward the ionosphere, providing energy for particle acceleration. In addition, these waves carry a field-aligned current that must close in the ionosphere. However, the ideal MHD Alfvén wave has no parallel electric field that can accelerate particles along the field to produce auroral emissions.

Observations of auroral particles at Earth show that the most common form of auroral acceleration is a nearly monoenergetic beam caused by a parallel potential drop (e.g., Gurnett and Frank 1973; Mozer et al. 1980). The electrons that produce the discrete aurora are associated with the acceleration of these electrons by electric fields parallel to the background magnetic field. The monoenergetic electrons are produced by a quasi-static parallel electric field that accelerates all the electrons to the same energy (e.g., Karlsson 2012). While the shear mode Alfvén wave can provide the energy to support such parallel electric fields (Song and Lysak 2001, 2006), it does not contain a parallel electric field itself.

The lack of parallel electric fields in the ideal MHD Alfvén wave suggests that modifications are necessary to provide these parallel electric fields. Early work by Hasegawa (1976) suggested that surface waves at the boundaries of the plasma sheet could mode convert into a kinetic Alfvén wave, with a perpendicular wavelength the order of the ion acoustic gyroradius $\rho_{s} = c_{s} /\Omega_{i}$, where $c_{s} = \left( {T_{e} /m_{i} } \right)^{1/2}$ is the ion acoustic speed and $\Omega_{i} = eB/m_{i}$ is the ion gyrofrequency. However, this work did not appear to place the parallel electric field in the low-altitude auroral region where auroral acceleration is most commonly observed. This was modified somewhat by Goertz and Boswell (1979) who pointed out that at lower altitudes, parallel electric fields can form when the perpendicular wavelength becomes comparable to the electron inertial length $\lambda_{e} = c/\omega_{pe}$ where *c* is the speed of light and $\omega_{pe} = (ne^{2} /\varepsilon_{0} m_{e} )^{1/2}$ is the plasma frequency. An early review of these initial measurements has been presented by Stasiewicz et al. (2000b).

However, in many cases, the aurora is associated with electrons having a broadband distribution in energy (e.g., Chaston et al. 2002a, b; Semeter and Blixt 2006). A broadband acceleration of these electrons would require that the parallel electric field would vary over the time it takes for the electrons to pass through the acceleration region. Observations (e.g., Mälkki et al. 1993) indicate that the parallel electric fields exist over a distance of roughly 1 *R*_E_ (1 *R*_E_ = 1 Earth radius = 6378 km). It is useful to note that a 100 eV electron travels about 1 *R*_E_/s, suggesting that fluctuations in the 1-s range are necessary to make these broadband distributions. Such fluctuations may be associated with the strong gradients in Alfvén speed $V_{A} = B/(\mu_{0} nm_{i} )^{1/2}$ caused by the rapid decrease of the plasma density above the ionosphere, forming a structure that has been called the “ionospheric Alfvén resonator” (IAR, Polykov and Rapaport 1981). Alfvén waves can be partially trapped in this resonator and will oscillate with its characteristic frequencies, which are in the 0.1–10.0 Hz range at Earth (e.g., Lysak 1991). The rapid fluctuations in the IAR can lead to a strong phase mixing that decreases the perpendicular wavelength so that parallel electric fields can form due to the electron inertial effects.

Therefore, understanding auroral particle acceleration then requires a number of ingredients: first, the generation of Alfvén waves that can carry field-aligned current and energy from the outer magnetosphere toward the ionosphere; second, the reduction of the perpendicular wavelength of Alfvén waves to form the kinetic Alfvén wave that carries a parallel electric field; and finally, the rapid fluctuations of the Alfvén waves that can lead to the broadband acceleration of electrons as is observed by satellites. In the following sections, we will first review the theory of the kinetic Alfvén waves along with relevant observations. Then, we will discuss the mechanisms that can lead to shorter perpendicular wavelengths, and finally, we will introduce the interactions with the ionosphere and discuss the role of the ionosphere and the ionospheric Alfvén resonator. We will conclude with some remarks on the application of kinetic Alfvén waves to auroral particle acceleration at Jupiter.

## Theory of the kinetic Alfvén wave

The seminal reference on the theory of the kinetic Alfvén wave as applied to auroral particle acceleration was work by Hasegawa (1976), who proposed that the mode coupling between an Alfvén surface mode and the kinetic Alfvén wave could lead to heating and auroral particle acceleration. This work built on previous work considering the effect of the kinetic Alfvén wave on plasma heating (e.g., Tataronis and Grossmann 1973; Hasegawa and Chen 1975). Hasegawa (1976) noted that an Alfvén surface wave between a plasma sheet with a mass density ρ and a vacuum was given by $\omega = \sqrt 2 k_{\parallel } V_{A}$, where the Alfvén speed is based on the plasma sheet parameters. Furthermore, using a two-fluid theory including hot electrons, he noted that in the bulk plasma the Alfvén wave dispersion relation becomes

1 $$\omega = k_{\parallel } V_{{\text{A}}} \sqrt {1 + k_{ \bot }^{2} \rho_{{}}^{2} }$$

Here, $\rho^{2} = \rho_{s}^{2} + (3/4)\rho_{i}^{2} ,$ where *ρ*_s_ is the ion acoustic gyroradius defined above and *ρ*_i_ is the ion gyroradius. The factor of 3/4 in this expression is due to a Bessel function expansion. This implies that the surface wave could couple to the kinetic Alfvén wave with a perpendicular wave number that satisfies $k_{ \bot } \rho = 1$. In this model, he also showed that the parallel electric field was given by

2 $$E_{\parallel } = k_{\parallel } k_{ \bot } \rho_{{\text{s}}}^{2} E_{ \bot } .$$

Note that in this expression, only the ion acoustic gyroradius *ρ*_s_ appears; the ion gyroradius does not affect the parallel electric field. This work was extended by Goertz and Boswell (1979), who noted that the parallel field implied by Eq. (2) for downward propagating Alfvén waves was actually in the wrong sense to accelerate electrons. They instead noted that the Hasegawa (1976) result only was correct in the regime where the plasma $\beta = 2\mu_{0} nT_{{\text{e}}} /B^{2}$ was greater than the electron-to-ion mass ratio, or equivalently, that the electron thermal speed was greater than the Alfvén speed. In the lower *β* regime, it is necessary to include the finite electron mass in the model, which leads to a modified dispersion relation:

3 $$\omega = \frac{{k_{\parallel } V_{{\text{A}}} }}{{\sqrt {1 + k_{ \bot }^{2} \lambda_{{\text{e}}}^{2} } }},$$

where *λ*_e_ is the electron inertial length defined above. In the Goertz and Boswell (1979) model, the parallel electric field pointed in the proper direction to accelerate electrons downward, consistent with the measurements of inward pointing perpendicular fields from the S3-3 satellite (Mozer et al. 1977).

A fully kinetic dispersion relation for the kinetic Alfvén wave was presented by Lysak and Lotko (1996) by solving the Vlasov–Maxwell equations for a Maxwellian plasma with *β* << 1. This calculation was simplified by considering low-frequency waves, $\omega \ll \Omega_{{\text{i}}}$, with large perpendicular wavenumber, $k_{ \bot } \rho_{{\text{s}}} \gg \beta^{1/2}$. Under these conditions, the fast mode wave, which has a dispersion relation $\omega^{2} = k^{2} V_{{\text{A}}}^{2} + k_{ \bot }^{2} c_{{\text{s}}}^{2}$, has a frequency greater than the ion gyrofrequency and so decouples from the shear mode. Due to this decoupling, the dispersion relation can be written as the determinant of a 2 × 2 matrix:

4 $$\det \left( {\begin{array}{*{20}c} {\varepsilon_{ \bot } - n_{\parallel }^{2} } & {n_{\parallel } n_{ \bot } } \\ {n_{\parallel } n_{ \bot } } & {\varepsilon_{\parallel } - n_{ \bot }^{2} } \\ \end{array} } \right) = 0$$

Here, the perpendicular and parallel dielectric constants are given by

5 $$\varepsilon_{ \bot } = 1 + \frac{{c^{2} }}{{V_{{\text{A}}}^{2} }}\frac{{1 - \Gamma_{0} \left( {\mu_{{\text{i}}} } \right)}}{{\mu_{{\text{i}}} }}$$

6 $$\varepsilon_{\parallel } = 1 + \frac{{\Gamma_{0} \left( {\mu_{{\text{e}}} } \right)}}{{k_{\parallel }^{2} \lambda_{{{\text{De}}}}^{2} }}\left( {1 + \xi Z\left( \xi \right)} \right)$$

In these expressions, $n_{ \bot ,\parallel } = k_{ \bot ,\parallel } c/\omega$ are the perpendicular and parallel indices of refraction, $\mu_{{\text{i,e}}} = k_{ \bot }^{2} \rho_{{\text{i,e}}}^{2}$ where *ρ*_i_ and *ρ*_e_ are the ion and electron thermal gyroradii, respectively, $\Gamma_{0} \left( \mu \right) = {\text{e}}^{ - \mu } I_{0} \left( \mu \right)$ is a modified Bessel function, $\lambda_{{{\text{De}}}} = (\varepsilon_{0} T_{{\text{e}}} /ne^{2} )^{1/2}$ is the electron Debye length, $\xi = \omega /k_{\parallel } a_{{\text{e}}}$ is the ratio of the parallel phase speed to the electron thermal speed and *Z* is the usual plasma dispersion function (Fried and Conte 1961). Under the assumptions that $V_{{\text{A}}} /c \ll 1$ and $k_{\parallel } \lambda_{{{\text{De}}}} \ll 1$, the first term in Eqs. (5) and (6) is negligible and the solution to the dispersion relation can be written as

7 $$\left( {\frac{\omega }{{k_{\parallel } V_{{\text{A}}} }}} \right)^{2} = \frac{{\mu_{{\text{i}}} }}{{1 - \Gamma_{0} \left( {\mu_{{\text{i}}} } \right)}} + \frac{{k_{ \bot }^{2} \rho_{{\text{s}}}^{2} }}{1 + \xi Z\left( \xi \right)}$$

Here, we have also assumed *μ*_e_ << 1 so the $\Gamma_{0} \left( {\mu_{{\text{e}}} } \right) \approx 1$. For all values of *μ*_i_, the first term on the right-hand side of Eq. (7) can be written using a Padé approximation (Johnson and Cheng 1997) $\mu_{{\text{i}}} /(1 - \Gamma_{0} (\mu_{{\text{i}}} )) \approx 1 + \mu_{{\text{i}}}$. (Note that this is a better approximation over the whole range of *μ*_i_ than the Bessel function expression used by Hasegawa 1976.) In the warm electron limit, where the electron thermal speed is greater than the Alfvén speed, *ξ* << 1 and the denominator of the second term is one, so we have

8 $$\left( {\frac{\omega }{{k_{\parallel } V_{{\text{A}}} }}} \right)^{2} = 1 + k_{ \bot }^{2} \left( {\rho_{{\text{s}}}^{2} + \rho_{{\text{i}}}^{2} } \right).$$

This expression extends Eq. (1) to include the finite ion gyroradius. On the other hand, when the electrons are cold, *ξ* >> 1 and $1 + \xi Z(\xi ) = 1 + \xi \left( { - 1/\xi - 1/2\xi^{3} } \right) = - 1/2\xi^{2}$. In this case, which can be called the inertial limit of the kinetic Alfvén wave, the dispersion relation becomes

9 $$\left( {\frac{\omega }{{k_{\parallel } V_{{\text{A}}} }}} \right)^{2} = \frac{{1 + k_{ \bot }^{2} \rho_{{\text{i}}}^{2} }}{{1 + k_{ \bot }^{2} \lambda_{{\text{e}}}^{2} }}.$$

A major difference between Eqs. (8) and (9) is that the perpendicular component of the group velocity is opposite to the direction of **k**_⊥_ in the inertial limit when the electron inertial length is greater than the ion gyroradius, while in the warm plasma limit, the group velocity is in the same direction. Gyrokinetic theories of the kinetic Alfvén wave that emphasize the finite ion gyroradius effects have been reviewed by Chen et al. (2021).

The polarization relations for this mode can be found by multiplying the dielectric tensor in Eq. (4) by the electric field components, which gives (Lysak 1998)

10 $$\frac{{E_{\parallel } }}{{E_{ \bot } }} = \frac{{n_{\parallel }^{2} - \varepsilon_{ \bot } }}{{n_{\parallel } n_{ \bot } }} = \left\{ {\begin{array}{*{20}c} { - \frac{{k_{\parallel } k_{ \bot } \rho_{{\text{s}}}^{2} }}{{1 + \mu_{{\text{i}}} }}} & {\beta > m_{{\text{e}}} /m_{{\text{i}}} } \\ {\frac{{k_{\parallel } k_{ \bot } \lambda_{{\text{e}}}^{2} }}{{1 + k_{ \bot }^{2} \lambda_{{\text{e}}}^{2} }}} & {\beta < m_{{\text{e}}} /m_{{\text{i}}} } \\ \end{array} } \right.$$

Here, the top line gives the warm plasma result, and the bottom line the result for the cold plasma. The ratio of the electric-to-magnetic field, which is an easily observable quantity, is given by

11 $$\frac{{E_{x} }}{{B_{y} }} = \frac{{n_{\parallel } }}{{\varepsilon_{ \bot } }} = \left\{ {\begin{array}{*{20}c} {V_{{\text{A}}} \frac{{1 + \mu_{{\text{i}}} }}{{\sqrt {1 + \mu_{{\text{i}}} + k_{ \bot }^{2} \rho_{{\text{s}}}^{2} } }}} & {\beta > m_{{\text{e}}} /m_{{\text{i}}} } \\ {V_{{\text{A}}} \sqrt {(1 + k_{ \bot }^{2} \lambda_{{\text{e}}}^{2} )(1 + \mu_{{\text{i}}} )} } & {\beta < m_{{\text{e}}} /m_{{\text{i}}} } \\ \end{array} } \right..$$

Again, the top and bottom lines give the warm and cold plasma results. Equation (11) can be used to verify the existence of the kinetic Alfvén wave, as was done with data from Freja (Stasiewicz et al. 2000a) and from FAST (Chaston et al. 2006).

**Fig. 1:**
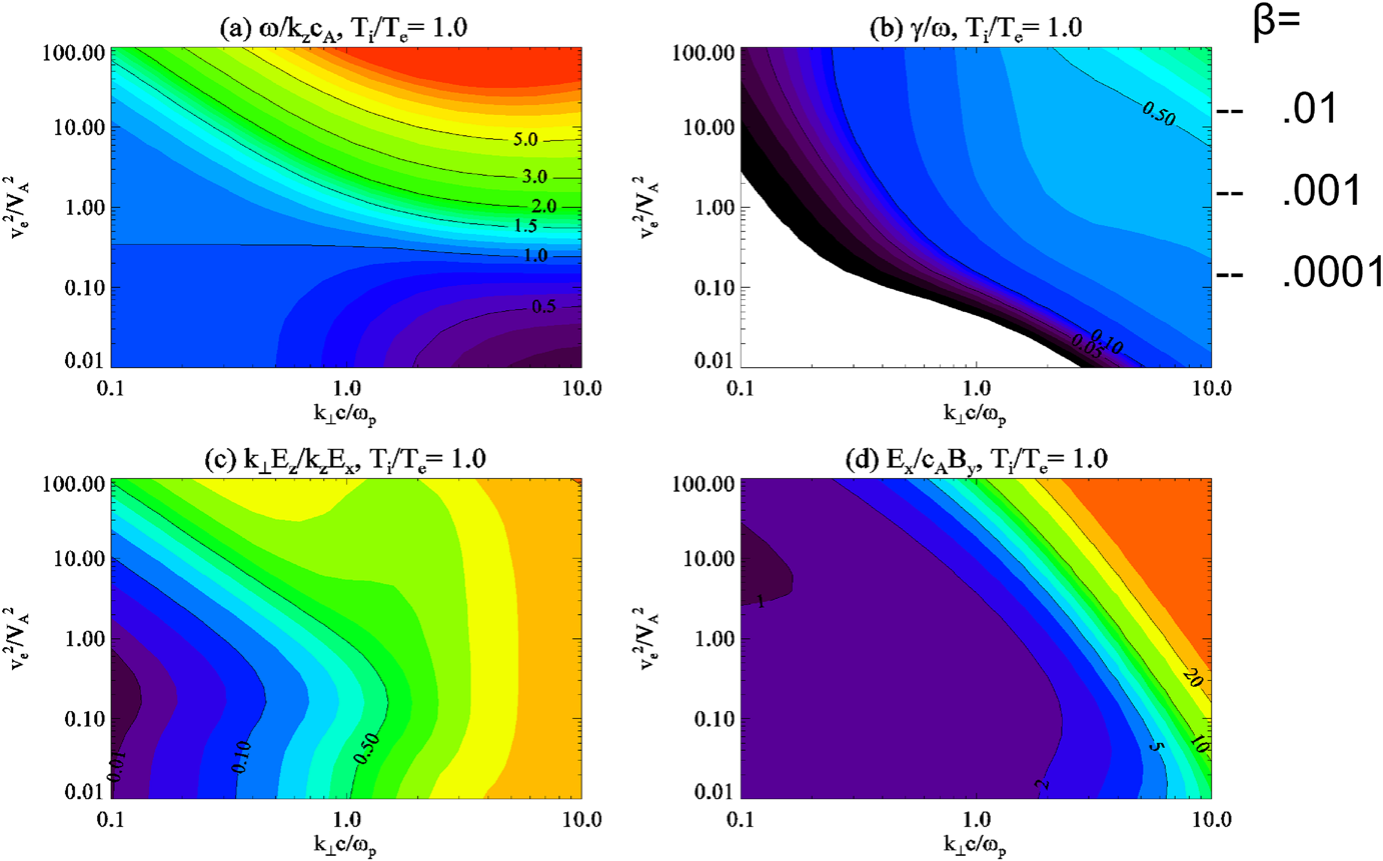
HYPERLINK "sps:id::fig1||locator::gr1||mediaobject::0"Dispersion relation for the kinetic Alfvén wave as a function of $k_{ \bot } c/\omega_{pe}$ and $v_{e}^{2} /V_{A}^{2}$. **a** Parallel phase velocity normalized by the Alfvén speed; **b** Landau damping rate normalized by wave frequency; **c** ratio of parallel electric field to the perpendicular electric field, normalized by the ratio of parallel to perpendicular wave numbers; **d** ratio of electric field to magnetic field normalized by the Alfvén speed. (after Lysak and Lotko 1996; Lysak 1998)

Solutions to the dispersion relation are shown in Fig. 1 (adapted from Lysak and Lotko 1996, and Lysak 1998), with each panel plotting various quantities as a function of perpendicular wavelength and the square of the ratio of the electron thermal speed to the Alfvén speed, which is proportional to the plasma *β*. Figure 1a shows the parallel phase velocity normalized to the Alfvén speed as calculated from Eq. (4). The characteristic features that the wave speed is greater than the Alfvén speed in the warm plasma regime and less than it in the cold plasma region can be seen. Figure 1b gives the Landau damping rate normalized to the wave frequency. This damping is strongest in the warm plasma regime and for large perpendicular wave number. Figure 1c gives the ratio of the parallel electric field to the perpendicular field, normalized to the ratio of the parallel to perpendicular wave numbers. It is worth noting that when this ratio is 1, the wave becomes quasi-electrostatic. Figure 1c shows that this occurs for large perpendicular wave number for all electron temperatures. Finally, Fig. 1d shows that electric-to-magnetic field ratio normalized to the Alfvén speed. As expected from Eq. (11), this ratio is one for small perpendicular wave numbers and increases at larger *k*_⊥_, consistent with the wave becoming approximately electrostatic.

While the approximations above are valid in many cases in the auroral zone, a few modifications are necessary in some cases. Near the peak of the Alfvén speed in the Earth’s auroral zone, the Alfvén speed may approach the speed of light. For example, near the FAST apogee at 4000 km, the magnetic field is about 0.1 G and the density can be as low as 1 cm^−3^ (e.g., McFadden et al. 1999), giving an Alfvén speed of 200,000 km/s, two-thirds of the speed of light. In Jupiter’s auroral zone, with magnetic fields about 20 times that of Earth and densities from 1 to 100 cm^−3^ (e.g., Elliott et al. 2021; Allegrini et al. 2021), the Alfvén speed as calculated by the usual formula becomes greater than *c*. In this case, the first term in Eq. (5) cannot be ignored, with the result that the Alfvén speed is replaced by

12 $$c_{{\text{A}}} = \frac{{V_{{\text{A}}} }}{{\sqrt {1 + V_{{\text{A}}}^{2} /c^{2} } }}$$

Here, we use the notation *c*_A_ to distinguish this speed from the non-relativistic Alfvén speed *V*_A_. Physically, this represents the fact that the field-aligned current of the Alfvén wave is closed by a polarization current in the wave front; in the low-density case, this current is replaced by the displacement current.

A more substantial correction is necessary when the wave frequency approaches the ion gyrofrequency. This situation arises often in lab plasma experiments; for example, in the Large Aperture Plasma Device (LAPD) at UCLA, experiments at up to half the gyrofrequency have been carried out (Gekelman et al. 1997). These authors have written the dispersion relation in the form

13 $$\left( {\frac{{k_{ \bot } c}}{{\omega_{{{\text{pe}}}} }}} \right)^{2} = Z^{\prime}\left( \xi \right)\left[ {\frac{{V_{{\text{A}}}^{2} }}{{a_{{\text{e}}}^{2} }}\frac{{\mu_{{\text{i}}} \left( {1 - \omega^{2} /\Omega_{{\text{i}}}^{2} } \right)}}{{1 - \Gamma_{0} \left( {\mu_{{\text{i}}} } \right)}} - \xi^{2} } \right].$$

In this expression, $Z^{\prime}(\xi ) = - 2(1 + \xi Z(\xi ))$ is the derivative of the plasma dispersion function. To reconcile this result with the discussion above, Eq. (5) must be modified to read (Lysak 2008)

14 $$\varepsilon_{ \bot } = 1 + \frac{{\omega_{{{\text{pi}}}}^{2} }}{{\omega^{2} }}\frac{\omega }{{k_{\parallel } a_{{\text{i}}} }}\sum\limits_{n = - \infty }^{\infty } {\frac{{n^{2} \Gamma_{n} \left( {\mu_{{\text{i}}} } \right)}}{{\mu_{{\text{i}}} }}Z\left( {\frac{{\omega - n\Omega_{{\text{i}}} }}{{k_{\parallel } a_{{\text{i}}} }}} \right)} \equiv 1 + \frac{{c^{2} }}{{V_{{\text{A}}}^{2} }}G\left( {\mu_{{\text{i,}}} \omega /\Omega_{{\text{i}}} } \right).$$

If the wave frequency is not too close to the ion cyclotron frequency so that the *Z* function can be expanded for large argument, the ± *n* terms can be combined to give

15 $$G\left( {\mu_{{\text{i}}} ,\omega /\Omega_{{\text{i}}} } \right) = \sum\limits_{n = 1}^{\infty } {\frac{{2\Gamma_{n} \left( {\mu_{{\text{i}}} } \right)}}{{\mu_{{\text{i}}} }}\frac{1}{{\omega^{2} - n^{2} \Omega_{{\text{i}}}^{2} }}} = \frac{{1 - \Gamma_{0} \left( {\mu_{{\text{i}}} } \right)}}{{\mu_{{\text{i}}} }} + \frac{{2\Gamma_{1} \left( {\mu_{{\text{i}}} } \right)}}{{\mu_{{\text{i}}} }}\frac{{\omega^{2} /\Omega_{{\text{i}}}^{2} }}{{1 - \omega^{2} /\Omega_{{\text{i}}}^{2} }}$$

Here, we have made use of Bessel function sum rules and, in the second step, we have assumed that the wave frequency can be neglected compared to the harmonics of the ion gyrofrequency in all but the *n* = 1 term. It should be noted that Gekelman et al. (1997) result is consistent with this expression for

16 $$G\left( {\mu_{{\text{i}}} ,\omega /\Omega_{{\text{i}}} } \right) = \frac{{1 - \Gamma_{0} \left( {\mu_{{\text{i}}} } \right)}}{{\mu_{{\text{i}}} \left( {1 - \omega^{2} /\Omega_{{\text{i}}}^{2} } \right)}}$$

Numerical results show that both (15) and (16) are good approximations to the full expression (14), although Eq. (15) is somewhat more accurate (Lysak 2008).

The kinetic Alfvén wave dispersion relation has been verified in laboratory experiments at the LAPD in the kinetic limit (Kletzing et al. 2003) and the inertial limit (Kletzing et al. 2010). These measurements had frequencies ranging from 0.29 Ω_i_ to 0.525 Ω_i_. They found good agreement with the dispersion relations, although the effects of collisions was included in the latter work. More recently, the predicted effects of kinetic Alfvén waves on the electron distribution were verified by Schroeder et al. (2017), and the acceleration of electrons at the Landau resonance was observed by Schroeder et al. (2021). While these experiments do not perfectly mimic the situation in space, the ability of the LAPD to model the general dimensionless parameters in the auroral zone has led to a further understanding of the kinetic Alfvén wave dispersion.

## Cross-scale coupling and kinetic Alfvén waves

**Fig. 2:**
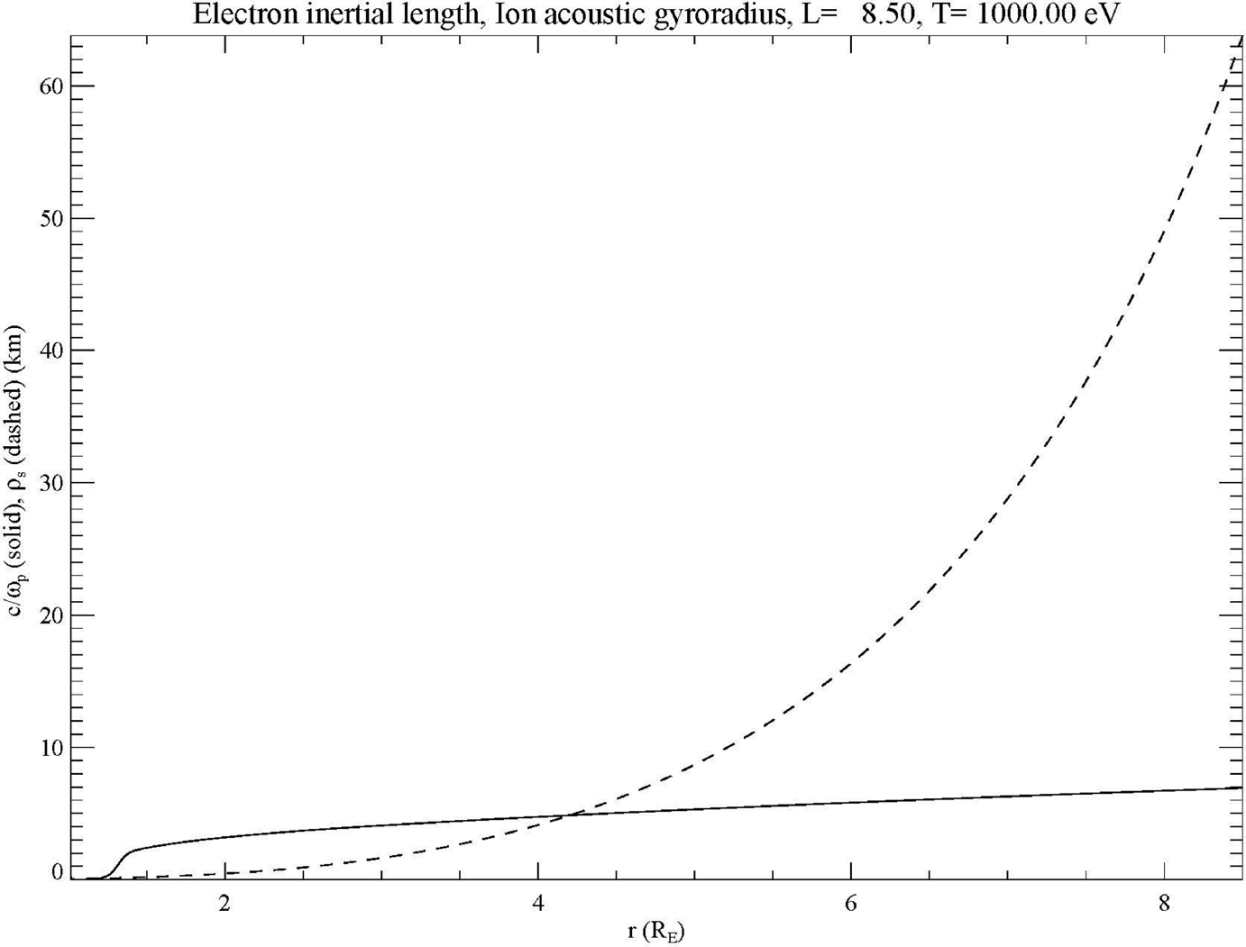
Plots of the electron inertial length (solid line) and ion acoustic gyroradius (dashed line) for an auroral field line in a model of Earth’s magnetosphere based on Van Allen probes data

The previous discussion has shown the need for kinetic Alfvén waves to have short perpendicular wavelengths to support parallel electric fields. However, many sources of field-aligned currents and Alfvén waves occur on larger scales. For example, Pi2 pulsations, which are ultra-low-frequency (ULF) Alfvén waves with periods of a few minutes (e.g., Keiling and Takahashi 2011) have been associated with bursty bulk flows in the tail (Angelopoulos et al. 1994; Kepko and Kivelson 1999; Kepko et al. 2001; Panov et al. 2010). However, these structures have scale lengths of 1–3 *R*_E_ in the near-Earth tail at about 10 *R*_E_ distance (Sergeev et al. 1996; Nakamura et al. 2004). Note that using a simple $\sqrt B$ scaling along the field line, these lengths would be 2000–6000 km at ionospheric altitudes. For comparison, Fig. 2 shows the two relevant scales, the electron inertial length and the ion acoustic gyroradius, for an auroral field line (*L* = 8.5) at Earth for the model shown in Fig. 12 of Takahashi et al. (2018) based on data from the Van Allen probes. This figure assumes an electron temperature of 1 keV. It can be seen that the electron inertial length is less than 10 km on this field line while the ion acoustic gyroradius reaches about 60 km. These scale lengths are, therefore, much less than the scales that would be produced in the generation of these waves. At Jupiter, Sulaiman et al. (2022) have shown that the density in the auroral zone can be as low as 10^−2^ cm^−3^, giving an inertial length of about 50 km. Saur et al. (2018) have shown that this inertial length maps to about 10,000 km, or about 0.14 *R*_J_, indicating that the electron inertial effect is dominant, at least within 30 *R*_J_ in the equatorial plane (here, Jupiter’s radius 1 *R*_J_ = 71,492 km). This is an order of magnitude smaller than a characteristic scale for turbulence in Jupiter’s magnetosphere of 1.7 *R*_J_ (Saur et al. 2002, 2003). These considerations imply that a mechanism for reducing the perpendicular scale must be operating at both Earth and Jupiter.

A number of possibilities for such a mechanism can be considered. First, it is well known that a turbulent cascade can transfer energy from large scales to small scales, following the seminal work of Kolmogorov (1941). Such turbulence has been studied in the solar wind (e.g., Tu and Marsch 1995; Bale et al. 2005), in the plasma sheets of Earth (e.g., Borovsky and Funsten 2003) and Jupiter (Saur et al. 2002, 2003), as well as in the Earth’s auroral zone (Chaston et al. 2008). In particular, Chaston et al. (2008, 2011) observed that the spectrum of magnetic fluctuations followed a Kolmogorov *k*^−5/3^ spectrum at low frequencies (i.e., large wavelengths) which breaks to a *k*^−7/3^ spectrum in the region where electron inertial effects become important. A similar break was seen in the solar wind turbulence by Bale et al. (2005). Thus, a turbulent cascade is a possible mechanism for cross-scale coupling in the auroral zone. However, although the visible aurora sometimes exhibits the complicated structure one would expect from turbulence, it is frequently organized into well-structured arcs, which would be difficult to understand if turbulence was the only process occurring.

**Fig. 3:**
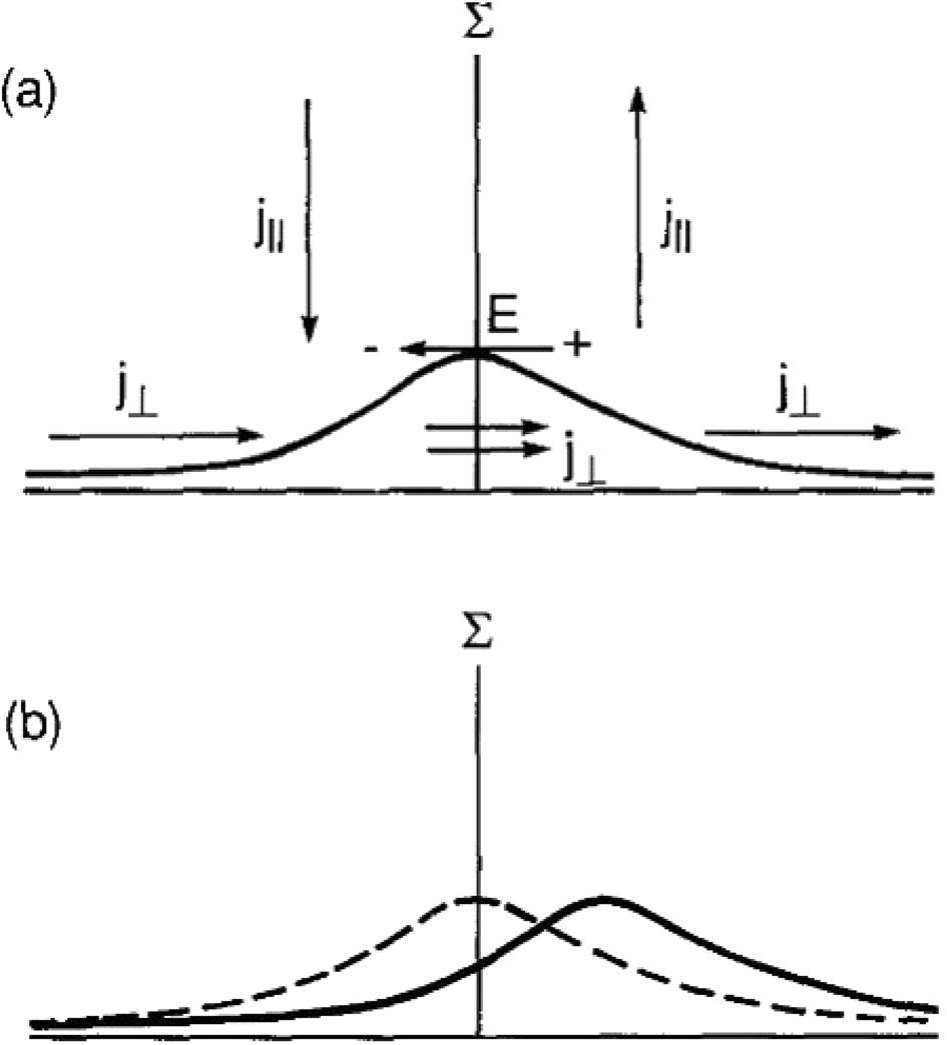
Schematic of feedback interaction in the ionosphere. **a** Plot of enhancement in Pedersen conductance (solid curve) showing the effect of enhanced current and reversed polarization electric field together with field-aligned currents that close the enhanced Pedersen current. **b** Effect of feedback, with the upward current region leading to increased Pedersen conductance due to precipitation and the downward current region having decreased Pedersen conductance due to electrons moving up the field line. Dashed curve is original profile (from panel **a**) and solid curve is result from feedback (Lysak 1990)

A second possibility for transferring energy to small scales occurs due to ionospheric feedback, introduced originally by Atkinson (1970) and further developed by Holzer and Sato (1973), Sato (1978) and Miura and Sato (1980). The basic premise behind this model is that in the presence of a background convection electric field, a localized conductivity enhancement due to electron precipitation will polarize, leading to field-aligned currents at the conductivity gradients. These field-aligned currents can then lead to additional precipitation that can in turn increase the conductivity. If the phase of this precipitation is such that it enhances the conductivity where it is already high, the resulting feedback can become unstable. Figure 3 (Lysak 1990) shows a simplified version of this interaction, based on a more detailed picture by Miura and Sato (1980). This figure shows a two-dimensional version of the instability where a conductivity enhancement leads to an increase in the background Pedersen current along with a reversed polarization electric field. The upward field-aligned current on the right side of the enhancement will be associated with additional precipitation; in addition, the downward current on the left side will be carried by upward electrons, depleting the density there. Thus, the conductivity enhancement tends to move in the direction of the background current.

**Fig. 4:**
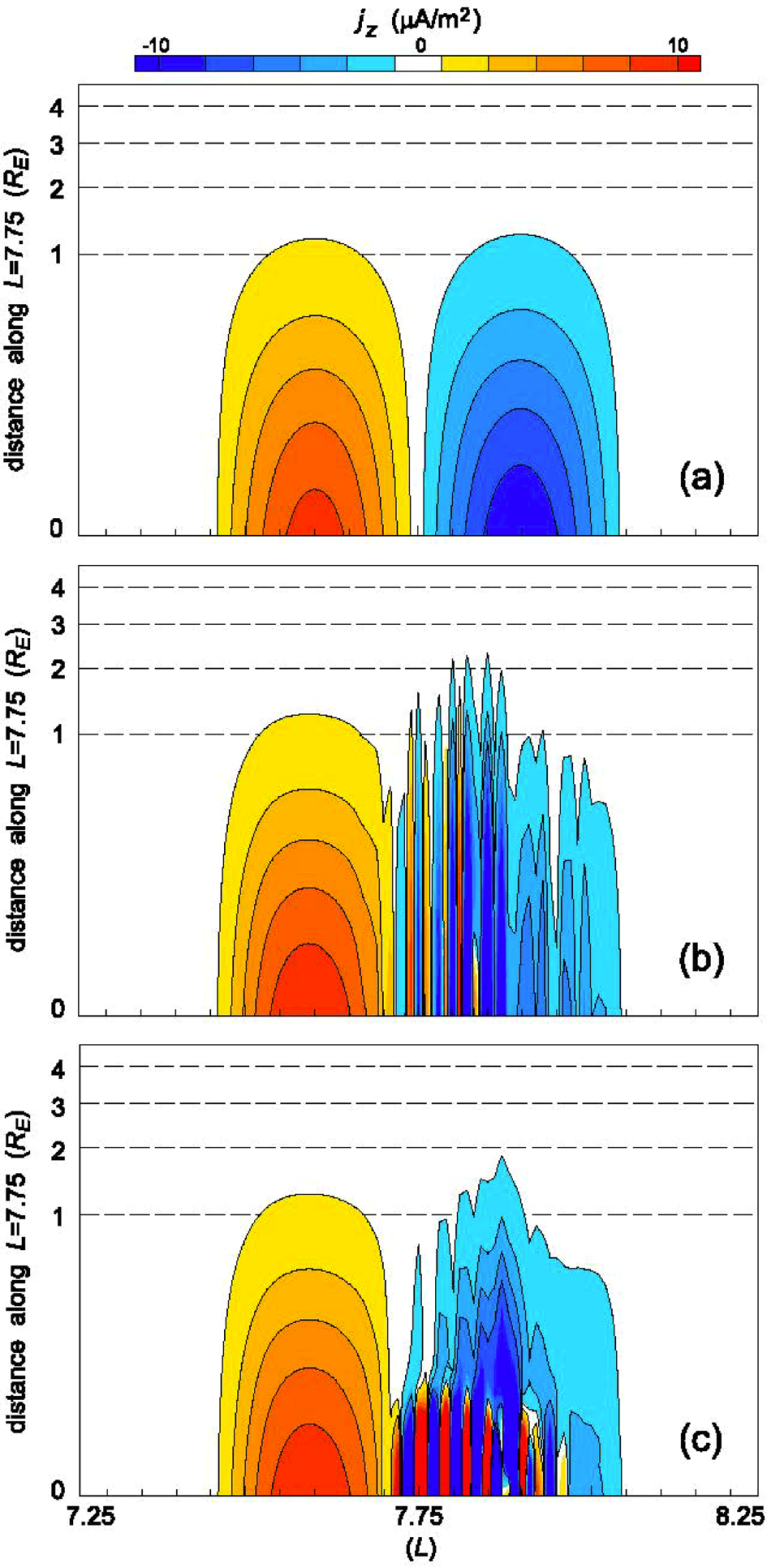
Simulations of feedback instability. Colors show field-aligned current density with yellow and red colors being upward current and green and blue being downward current: **a** simulation with no conductivity variation in the ionosphere; **b** simulation including ionospheric feedback for a field line resonant wave; **c** simulation including the ionospheric Alfvén resonator showing enhanced structuring at the lowest altitudes (Streltsov and Lotko 2008)

Early work on the ionospheric feedback instability considered the magnetospheric response to be characterized by a field line impedance (e.g., Miura and Sato 1980), while later work has considered this instability in the presence of field line resonances (Lu et al. 2007; Streltsov and Lotko 2004, 2008; Watanabe 2010, 2014) and in the ionospheric Alfvén resonator as described more completely below (Lysak 1991; Lysak and Song 2002). These simulations indicate that the feedback instability leads to very narrow current structures, apparently limited only by collisional effects in the ionosphere. Figure 4 (Streltsov and Lotko 2008) shows an example of the current structuring in regions of downward background current. Figure 4a shows the current structure without the feedback instability, with yellow and red colors indicating upward current and blue and green colors downward current. Without the feedback interaction, both current structures simply reflect the input pulse. Figure 4b shows the result when the feedback interaction is turned on. It can be clearly seen that the downward current region filaments into many smaller structures. It should be noted that the higher background conductivity in the upward current region acts to suppress the instability due to a higher recombination coefficient (Lysak and Song 2002). Figure 4c shows the modification when the ionospheric Alfvén resonator is turned on. In this case, the currents are most intense close to the ionosphere, as will be discussed below.

Most of the previous work on the feedback instability has considered only a height-integrated slab ionosphere. This restriction was relaxed by Sydorenko and Rankin (2017) who considered a full height-resolved ionospheric model. Their conclusion was that the shear in the Pedersen drift velocity due to the variation of Pedersen conductivity with height stabilized the feedback instability. This conclusion was disputed by Watanabe and Maeyama (2018), who performed an eigenmode analysis of the feedback instability in the presence of a height-resolved and found it to be unstable, although with somewhat lower growth rates than in the height-integrated case. They suggested that the negative results of Sydorenko and Rankin (2017) might be due to the fact that they solved an initial value problems where the initial conditions were far from the unstable eigenmodes of the system. This matter remains to be resolved.

The presence of the feedback instability has been challenging to confirm observationally. Results from the Auroral Currents and Electrodynamics Structure (ACES) sounding rocket campaign showed filamentary current structure in the downward current region, consistent with theory (Cohen et al. 2013). The Magnetosphere–Ionosphere Coupling in the Alfvén resonator (MICA) sounding rocket observed strong, small-scale field-aligned current associated with conductivity gradients (Lynch et al. 2015), again consistent with the overall picture of feedback. It should be noted that it may not be strictly necessary for the feedback interaction to go unstable for there to be an effect on the structure of the auroral field-aligned currents. However, the MICA results also indicated that the feedback interaction may not be solely responsible for this structuring, suggesting that the neutral winds may have a significant impact in the transition region between upward and downward currents.

A final mechanism for the production of waves with small perpendicular wavelengths is the process of phase mixing. Phase mixing occurs when the wave speed has a gradient across the magnetic field, so that waves on adjacent field lines propagate at slightly different speeds. This leads to a shearing of the phase fronts of the wave, producing a smaller perpendicular wavelength (Burghes et al. 1969; Heyvaerts and Priest 1983). Phase mixing was invoked by Hasegawa (1976) to discuss the production of kinetic Alfvén waves in the plasma sheet boundary layer. This effect is particularly pronounced for waves that form standing modes on closed field lines, often called field line resonances (FLRs), which form a continuous spectrum in the magnetosphere due to differences in density, magnetic field, and field line length (e.g., Cummings et al. 1969; Rankin et al. 2021). It has long been recognized that fast mode waves, for example, generated at the magnetopause, can propagate to the point where the wave frequency matches the characteristic of a field line and convert to shear Alfvén waves, a process called resonant mode conversion (Dungey 1954; Tamao 1964). The theory of FLRs was verified by Samson (1972) who showed that the polarization of the resonances was consistent with their excitation at the magnetopause. A more complete theory in a box model geometry was introduced by Zhu and Kivelson (1988).

Phase mixing provides a natural mechanism for the production of small perpendicular wavelength Alfvén waves in the magnetosphere. The association of these waves with auroral particle acceleration was made by Samson et al. (1991, 1996). Modeling of this process was performed in a box model by Rankin et al. (1993, 1995) and Streltsov and Lotko (1995) and in a dipole model by Streltsov and Lotko (1997), Streltsov et al. (1998) and Rankin et al. (1999a). These models verified the dispersion relations of the kinetic Alfvén wave as described above, showing the reversal of the perpendicular group velocity in the inertial limit. This aspect was emphasized by Streltsov and Lotko (1995, 1997), who showed that for a particular density distribution, the displacement due to the dispersion in the warm plasma limit could directly balance the opposite displacement in the cold plasma limit, potentially leading to a buildup of the wave amplitude.

While phase mixing can occur throughout the magnetosphere, it can be especially effective in regions of strong plasma density gradients, such as at the plasma sheet boundary layer (PSBL) (Lysak and Song 2011) or at the plasmapause (Streltsov and Mishin 2020). The effect of phase mixing at the PSBL was observed by the Polar satellite (Wygant et al. 2002), which showed strong kinetic Alfvén waves at 4–6 *R*_E_ together with an electron distribution that showed evidence of being accelerated in both directions by the parallel electric field of the wave. By comparing the *E*/*B* ratio with the theoretical dispersion relation (Lysak and Lotko 1996), they were able to estimate the perpendicular wavelength and the plasma *β* by comparing with the results shown in Fig. 1. This allowed for an estimate of the parallel potential in the wave that was consistent with the energy of the bidirectional electrons observed. An interesting aspect of these observations was that there was a tail in the electron distribution in the direction toward the Earth, in the same direction as the Poynting flux of the wave. This was interpreted as the result of acceleration of the electrons at the Landau resonance.

The generation of kinetic Alfvén waves in the plasma sheet can be a consequence of substorm processes in the tail. Kinetic Alfvén waves can be generated directly by reconnection processes (Shay et al. 2011; Sharma Pyakurel et al. 2018). Alternatively, the fast flows produced by reconnection, often cited as the source of Pi2 pulsations (Kepko and Kivelson 1999; Panov et al. 2014), can excite fast mode waves that propagate to the plasma sheet boundary layer and mode convert to kinetic Alfvén waves (Chi et al. 2009; Lysak et al. 2009; Lysak and Song 2011). One issue is that the small perpendicular wavelength of the waves can lead to Landau damping of the waves before they can reach the ionosphere. However, this effect can be balanced by the phase mixing process, which can continually regenerate kinetic Alfvén waves with small perpendicular wavelength.

Modeling of the direct acceleration of auroral electron by waves at the PSBL was presented by Watt and Rankin (2007, 2009, 2010). They demonstrated that the combined processes of electron trapping and acceleration at the Landau resonance can produce accelerated electron fluxes consistent with the observations of Wygant et al. (2002). Electron energization in field line resonances was also modeled using a hybrid approach that coupled a two-fluid model with a kinetic description of the accelerated electrons both at Earth (Damiano and Wright 2008; Damiano and Johnson 2012; Damiano et al. 2019a, b) and at Jupiter (Damiano et al. 2019a, b). This work concentrated on the warm plasma limit, and showed a monoenergetic acceleration of electrons coupled with the mirror force. This monoenergetic acceleration occurs since the periods of the field line resonances are a few minutes or more, much less than the electron transit time along auroral field lines (e.g., a 100 eV electron travels about 1 *R*_E_/*s*, and so could travel 60 *R*_E_ in a minute). This would imply that the field line resonance wave field would be essentially static over the time it takes for an electron to move along the field line. Thus, these electrons would all receive the same amount of acceleration, possibly producing a monoenergetic peak in the electron distribution. While such distributions are indeed observed, the broadband acceleration of auroral electrons requires a more dynamic acceleration mechanism. Another factor is that the broadband electron distributions are often very narrow in pitch angle (McFadden et al. 1986) indicating that these electrons are ionospheric in origin and have been accelerated at low altitudes. These considerations suggest that the interaction of kinetic Alfvén waves with the ionosphere is an important aspect of the auroral acceleration process.

## The ionospheric Alfvén resonator

The discussion above regarding the dispersion relation for kinetic Alfvén waves strictly applies only in a uniform plasma in which the Alfvén waves are traveling in a single direction. However, auroral field lines are terminated in the ionosphere, which closes the field-aligned current of the Alfvén wave and leads to its reflection. In the simplest case of a uniform flux tube terminated by an ionosphere with constant Pedersen conductance, the reflection coefficient for an Alfvén wave can be written as (Scholer 1970; Mallinckrodt and Carlson 1978)

17 $$R = \frac{{E_{{{\text{ref}}}} }}{{E_{{{\text{inc}}}} }} = \frac{{\Sigma_{{\text{A}}} - \Sigma_{{\text{P}}} }}{{\Sigma_{{\text{A}}} + \Sigma_{{\text{P}}} }}.$$

In this expression, $\Sigma_{{\text{A}}} = 1/\mu_{0} V_{{\text{A}}}$ is often called the Alfvén conductance and Σ_P_ is the Pedersen conductance. If the kinetic corrections to the Alfvén wave are included, the Alfvén speed in the Alfvén conductance is replaced by the *E/B* ratios given by Eq. (11). For both the Earth and Jupiter, the inertial limit of the kinetic Alfvén wave is most important near the ionosphere where the magnetic field is strong and the plasma is relatively cold so that the electron thermal speed is much less than the Alfvén speed (Lysak and Carlson 1981; Saur et al. 2018; Lysak et al. 2021). In this case, the observed electric and magnetic fields are the result of interference between the incident and reflected waves, leading to a ratio (Knudsen et al. 1992; Miles et al. 2018)

18 $$\frac{{E_{x} }}{{B_{y} }} = V_{A} \frac{{1 - R\,{\text{e}}^{{ - 2i\omega z/V_{{\text{A}}} }} }}{{1 + R\,{\text{e}}^{{ - 2i\omega z/V_{{{\text{A}} + }} }} }}$$

Here, *R* is the reflection coefficient from Eq. (17) and *z* is the altitude above the ionosphere. This ratio oscillates between $1/\mu_{0} \Sigma_{{\text{P}}}$ and $\mu_{0} V_{{\text{A}}}^{2} \Sigma_{{\text{P}}}$ as a function of altitude.

**Fig. 5:**
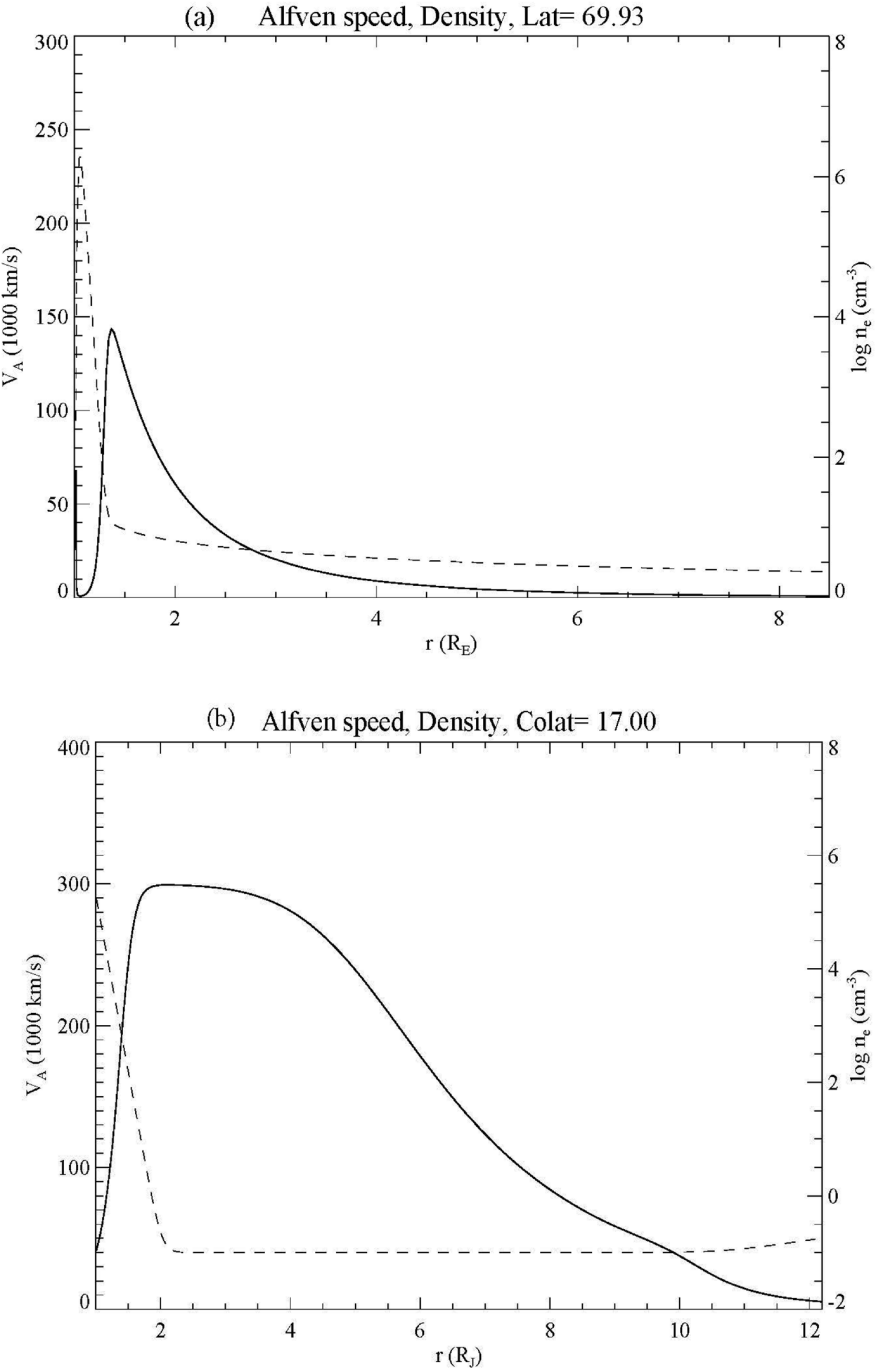
Alfvén speed (solid) and density (dashed) profiles for auroral field lines at **a** Earth, and **b** Jupiter

However, in general, the auroral flux tube above the ionosphere is not uniform. Since the plasma density decreases almost exponentially above the ionosphere while the magnetic field decreases more slowly, the Alfvén speed rises dramatically above the ionosphere. Figure 5a shows a typical density and magnetic field profile for Earth (adapted from Lysak et al. 2013) while Fig. 5b shows a corresponding profile from Jupiter (Lysak et al. 2021). Both cases show sharp increases in the Alfvén speed above the ionosphere, reaching the speed of light in the case of Jupiter and almost half the speed of light for Earth. This creates a virtual resonant cavity known as the ionospheric Alfvén resonator (IAR), first noticed in the spectral resonance signature from ionospheric heating experiments (Polykov and Rapaport 1981). Subsequent theoretical studies have explored the structure of this resonator (Trakhtengertz and Feldstein 1984; Lysak 1991, 1993; Lysak and Song 2008; Sydorenko et al. 2008; Lysak et al. 2013; Woodroffe and Lysak 2012) and experimental evidence for the IAR has been found from sounding rockets, ground magnetometers and low-Earth orbiting satellites (Cohen et al. 2013; Hebden et al. 2005; Hirano et al. 2005; Lynch et al. 2015; Miles et al. 2018; Pakhotin et al. 2018).

A simple theoretical model of the IAR was presented by Lysak (1991), based on an Alfvén speed profile first introduced by Trakhtengertz and Feldstein (1984):

19 $$V_{{\text{A}}}^{2} (z) = \frac{{V_{{{\text{AI}}}}^{2} }}{{\varepsilon^{2} + e^{ - z/h} }}$$

Here, *V*_AI_ is the Alfvén speed in the ionosphere, *ε* = *V*_AI_/*V*_AM_ where *V*_AM_ is the magnetospheric Alfvén speed and *h* is the density scale height. Solutions to the wave equation using this profile can be written in terms of Bessel functions with the argument $x = x_{0} {\text{e}}^{ - z/2h}$ where $x_{0} = 2h\omega /V_{{{\text{AI}}}}$. When coupled to the ionosphere, the eigenfrequencies of this resonator can be expressed as *ω*_*n*_ = ξ_*n*_*V*_AI_/2*h*, where in the limit that *α* = *μ*_0_*V*_AI_Σ_P_ >  > 1, *ξ*_*n*_ is the *n*th zero of the zeroth order Bessel function, 2.4, 5.5, 8.6,… These frequencies are in the 0.1–10.0 Hz range for typical ionospheric parameters. Note that the electron inertial corrections can be included by dividing the Alfvén speed in Eq. (19) and in the definition of the eigenfrequencies by $\sqrt {1 + k_{ \bot }^{2} \lambda_{e}^{2} }$ Somewhat surprisingly, the IAR at Jupiter also has the frequencies in this range: the Alfvén speed is much greater at Jupiter but so is the ionospheric scale height. More details about the dispersion relation and structure of these modes was presented by Lysak and Yoshikawa (2006), Lysak et al. (2021) and Lysak (2022).

**Fig. 6:**
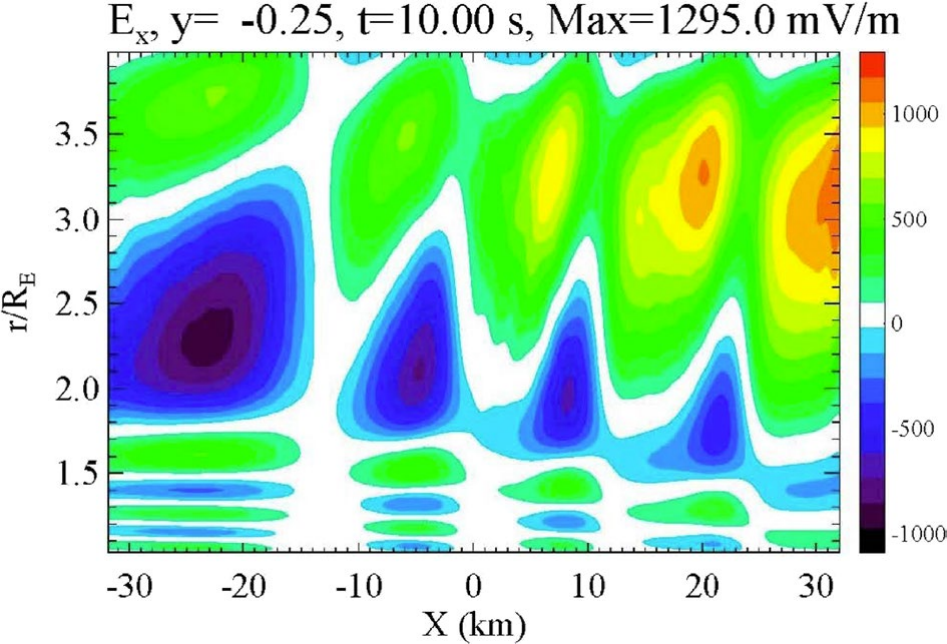
Electric fields produced by a 1 Hz pulse uniform in the *x* direction but with a density gradient with higher density on the left-hand side of the figure. The wave breaks up into filaments and shows the multiple nodes indicating modes of the ionospheric Alfvén resonator (Lysak and Song 2011)

The higher frequency of the waves in the IAR implies that processes such as phase mixing and ionospheric feedback can occur more rapidly than for lower frequency waves such as FLRs. For example, Fig. 6 (from Lysak and Song 2011) shows a simulation of the lower parts of the flux tube that was initialized with a 1 Hz wave that is uniform in the perpendicular direction. The background plasma model has a slight gradient in the density and thus the Alfvén speed, with higher Alfvén speeds on the right-hand side of the figure. After 10 s of simulation time, this pulse has broken up into structures with about a 10-km periodicity. In addition, the multiple mode structures of the IAR can be seen with different harmonics being excited on each field line due to the different density distributions.

**Fig. 7:**
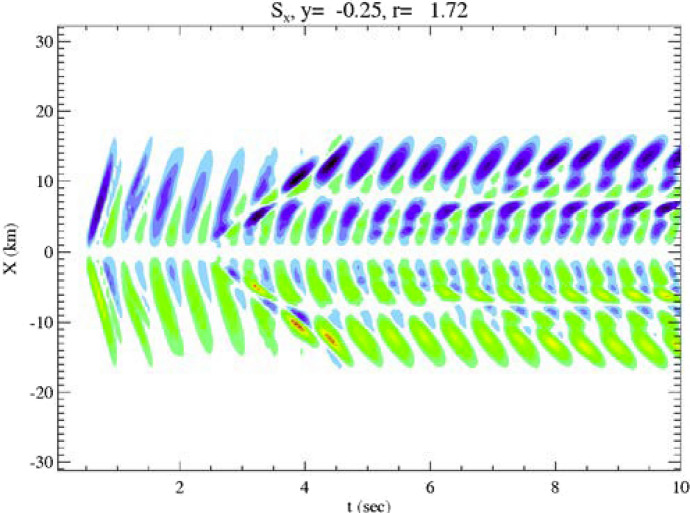
Horizontal component of the Poynting flux at a radial distance of 1.72 *R*_E_. The green and red colors represent positive Poynting flux (upward in the figure) while blue indicates negative Poynting flux (downward). Note that although the phase fronts propagate away from the central line, the Poynting flux is inward, consistent with the inertial limit of the dispersion relation (Lysak and Song 2008)

The effect of electron inertia is illustrated in Fig. 7 (Lysak and Song 2008) which shows a snapshot of the perpendicular Poynting flux from a run in which a pre-existing density cavity is imposed, following observations from the FAST satellite by Chaston et al. (2006). The vertical axis in this figure shows the direction perpendicular to the background field while the horizontal axis is time. Green and red colors indicate Poynting flux toward the top of the figure, while blue and purple show Poynting flux toward the bottom. The effect of finite perpendicular wavelength can be seen. In addition, the “backward” nature of the wave can be seen in the fact that the phase fronts are moving away from the central field line while the Poynting flux is inward. After about 3 s, the wave has reflected from the ionosphere, which can be seen in the change in slope of the phase fronts. This illustrates the interference between up and down going waves associated with enhanced phase mixing.

**Fig. 8:**
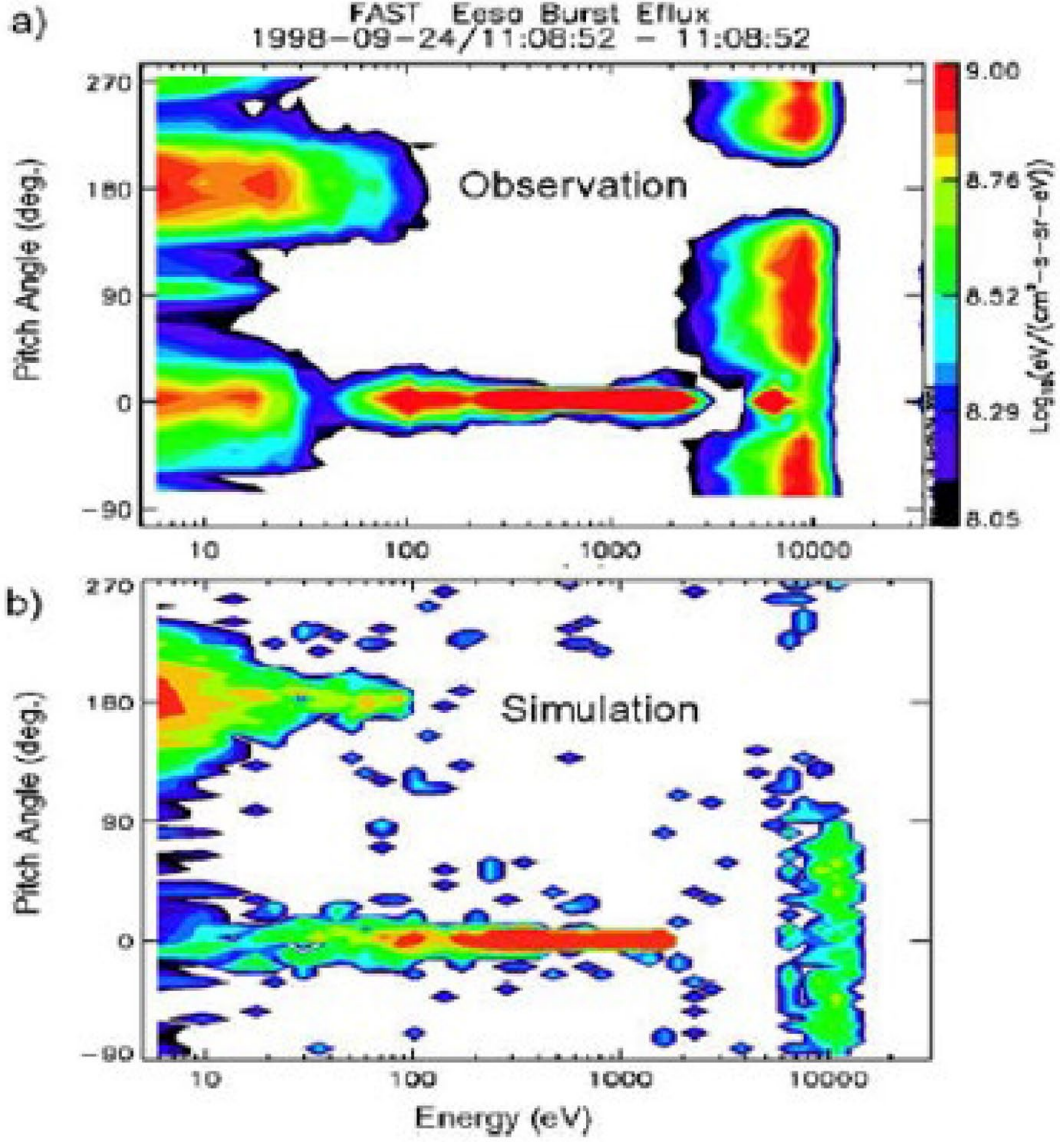
**a** Observations from the FAST satellite of an electron distribution plotted as a function of energy (horizontal) and pitch angle (vertical). The vertical bar at 5000–10,000 eV represents a monochromatic acceleration while the horizontal bar at zero pitch angle indicates the broadband electrons. Note the counter streaming at 180° pitch angle; **b** test particle simulation of the electron acceleration in a simulation of the kinetic Alfvén wave, showing many of the same features as the observation (Chaston et al. 2002a)

Acceleration of electrons in the IAR has largely been studied through test particle simulations, following original work by Thompson and Lysak (1996), who modeled the electron acceleration based on the parallel electric fields in a simulation of inertial Alfvén waves. These results showed that quasi-periodic bursts of field-aligned electrons, with periods of around 1 s, were produced by the Alfvén waves. A similar model was developed and applied to observations from the FAST satellite by Chaston et al. (2002a, b). Figure 8 (from Chaston et al. 2002a) compares observations from FAST (Fig. 8a) with test particle simulations (Fig. 8b) in a format where the horizontal axis is energy and the vertical axis is pitch angle. In this example, both monoenergetic acceleration, here indicated by a vertical bar at 5–10 keV, is seen together with a broadband distribution centered on zero pitch angle (downgoing), with a weaker emission at 180°, indicating a counter-streaming population.

**Fig. 9:**
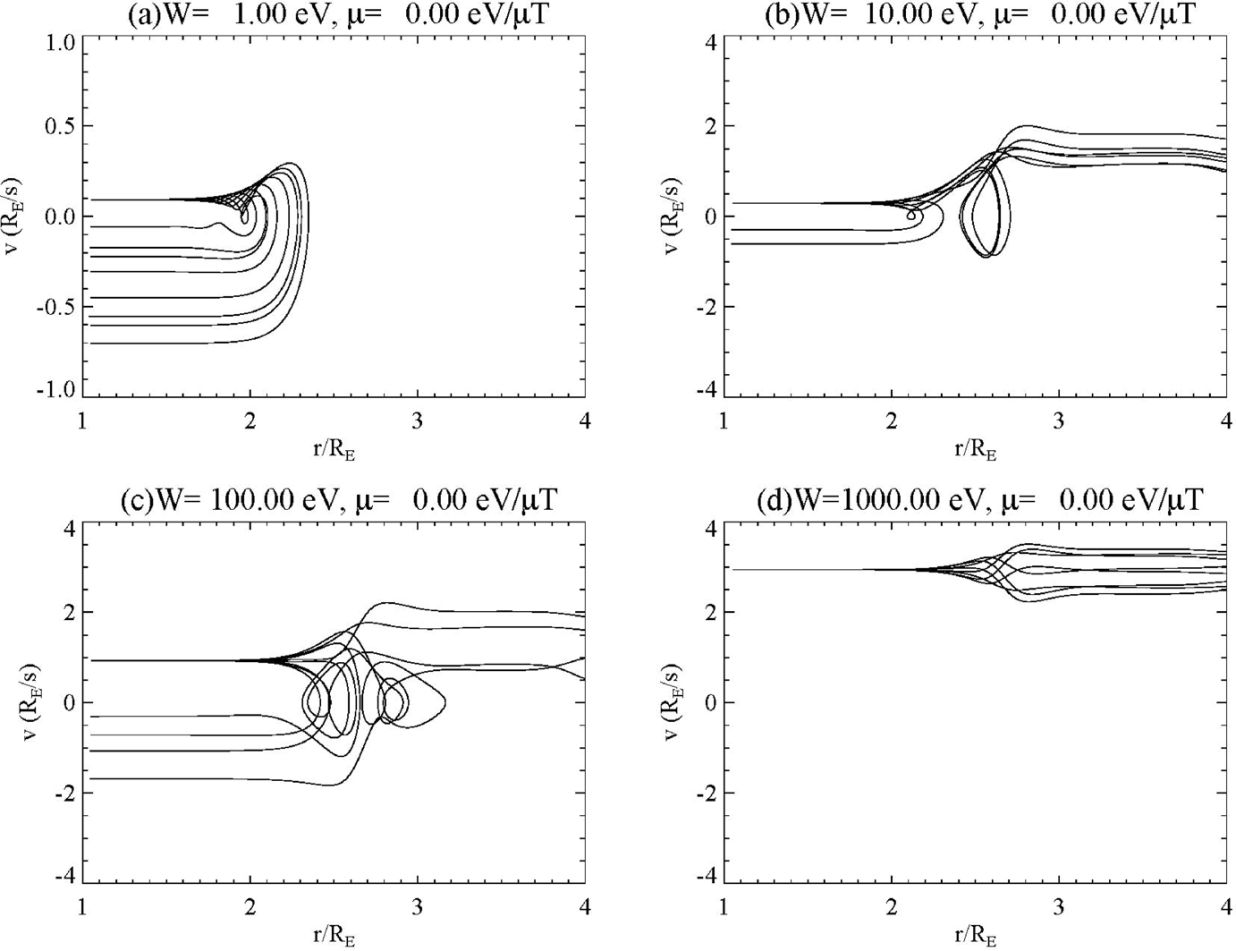
Ionosphere trajectories for electrons launched at 8 different phases of a monochromatic Alfvén wave at 0.22 Hz. **a** Initial energy *W* = 1 eV, with magnetic moment *μ* = 0; **b** *W* = 10 eV, *μ* = 0; **c** *W* = 100 eV, *μ* = 0; **d** *W* = 1 keV, *μ* = 0. (Lysak and Song 2005)

A test particle model based on a kinetic equilibrium model of an auroral flux tube was presented by Lysak and Song (2005). The parallel electric field structure in the kinetic Alfvén wave was determined by the solution of a self-consistent integral equation describing kinetic Alfvén waves in the IAR (Lysak and Song 2003) based on a similar model for field line resonant fields introduced by Rankin et al. (1999b) and Tikhonchuk and Rankin (2000, 2002). Figure 9 shows the trajectories for electrons initiated at the ionosphere with energies of 1, 10, 100 and 1000 eV with zero magnetic moment in the presence of a wave at 0.22 Hz, which is the fundamental IAR mode for the density profile used. Note that for this run, the total integrated parallel electric field amplitude is 300 V and is localized in the 2–3 *R*_E_ altitude range. Each panel in the figure has 8 curves, corresponding to electrons launched from the ionosphere at different phases of the wave field. The 1 eV electrons are all reflected by the parallel electric field of the wave, but receive a different amount of energization with the maximum being a speed of 0.7 *R*_E_/*s*, which is about 50 eV for an electron. Thus, the acceleration of the cold ionospheric population will result in a broadband distribution of electrons. For initial energies of 10 eV and 100 eV, some of the electrons are reflected but some escape the region of parallel electric field, while the 1 keV electrons all escape and are heated slightly by passing through the parallel electric field region. It should be noted that these runs used a very weak Alfvén wave with a Poynting flux of 0.65 mW/m^2^, lower than typical auroral zone waves. Thus, the energization would be stronger for waves with observed amplitudes of up to 100 mW/m^2^ (Wygant et al. 2002).

**Fig. 10:**
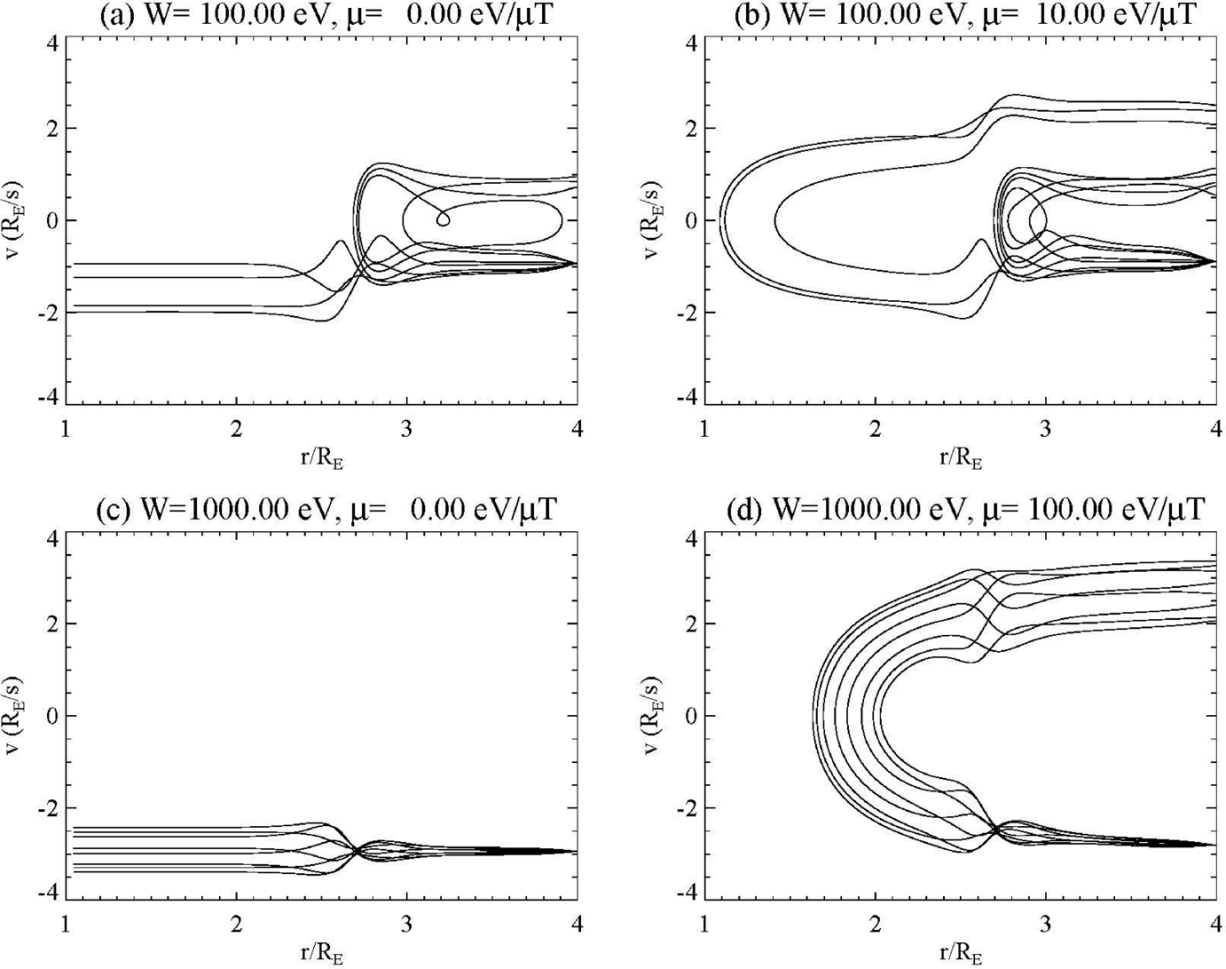
Trajectories of electrons from the magnetosphere at 8 phases of the wave as in Fig. 9. **a** *W* = 100 eV, *μ* = 0; **b** *W* = 100 eV, *μ* = 10 eV/μT; **c** *W* = 1000 eV, *μ* = 0; **d** *W* = 1000 eV, *μ* = 100 eV/μT (Lysak and Song 2005)

Figure 10 shows the trajectories of particles originating in the magnetosphere, with Fig. 10a, b showing results for electrons having initial energies of 100 eV with magnetic moments of 0 and 10 eV/μT, respectively, while Fig. 10c, d show 1 keV electrons with magnetic moments of 0 and 100 eV/μT. The cases with 0 magnetic moment are partially reflected with some particles precipitating, similar to the ionospheric particles. At 100 eV, some of the electrons are reflected while at 1 keV, all electrons pass through the region of parallel electric field, with some heating. However, for the cases with finite magnetic moment, magnetic mirroring becomes important. Note that both Fig. 10b, d have *W*/*μ* = 10 μT, indicating that in the absence of parallel electric fields, they would mirror where the magnetic field is 10 μT, or at 1.79 *R*_E_. The 1 keV electrons do in fact mirror in this region, while the 100 eV electrons mirror above or below this region depending on the phase of the wave when the particles are injected.

## Discussion and conclusions

The considerations above show the ability of kinetic Alfvén waves to produce the parallel electric fields needed to account for the broadband electron distributions often seen in conjunction with aurora arcs. The connection of kinetic Alfvén waves with the aurora at Earth has been well established with observations from the FAST satellite (e.g., Chaston et al. 2007) and, more recently, from the Swarm constellation of satellites (Wu et al. 2020). A recent review of dynamic aurora phenomena has also been given by Kataoka et al. (2021), while the quasi-static acceleration at Earth generally produces more energetic electrons and larger energy fluxes, as we have seen above these can also be Alfvénic in character, due to the low frequencies of field line resonances.

The observational situation is less clear at Jupiter, with auroral zone data only being available recently from the Juno satellite. One of the early observations from Juno was the relative importance of broadband acceleration of auroral electrons (Mauk et al. 2017), which were shown to have a stronger energy flux than for monoenergetic electron distributions. Clear observations of Alfvén waves are present on the Io flux tube (Gershman et al. 2019; Sulaiman et al. 2020), along with broadband acceleration of electrons (Szalay et al. 2018) and ions (Szalay et al. 2020).

The main auroral region at Jupiter is more complex. Mauk et al. (2020) have organized the observations of the main aurora into three regions, the Diffuse Auroral region, a Zone I upward field-aligned current regions showing mostly downward electron acceleration and a Zone II, which is largely downward currents but has bidirectional electron acceleration. All these regions can exhibit broadband electron distributions. However, the only region showing strong Alfvén wave power is in the diffuse auroral region.

This leads to a number of possibilities. One is that the broadband acceleration may be associated with whistler mode waves, which have been observed in these regions (e.g., Kurth et al. 2018). These authors observed an inverted-V event during the 7th Juno perijove crossing in which the peak of the inverted-V transitioned into a broadband distribution accompanied by strong electromagnetic wave activity above the ion gyrofrequency. Another possibility lies in the observation that the plasma density decreases rapidly going from the diffuse aurora region to Zone I (Sulaiman et al. 2021, 2022), reaching densities as low as 0.01 cm^−3^. Such low plasma densities imply that the Alfvén speed will approach the speed of light (see Eq. (12)). This has a number of consequences. First of all, low densities increase the electron inertial length, which can be estimated as 5 km/$\sqrt n$, where *n* is in cm^−3^. This will enhance the parallel electric field in any waves present. The other consequence is that low densities make the magnetic field perturbation weak. For a given Poynting flux *S*, the magnetic field perturbation is $b = \sqrt {\mu_{0} S/V_{A} }$ and so high Alfvén speeds imply small magnetic fields. Another possibility is that the acceleration of electrons by Alfvén waves at higher altitudes than the Juno orbit causes the waves to dissipate before reaching the satellite. This may be particularly important in Zone I where the broadband electron acceleration is mainly downward.

In summary, kinetic Alfvén waves play a critical role in the acceleration of auroral electrons both at Earth and at Jupiter. The Poynting flux associated with these waves can provide the energy for particle energization in the auroral zones. The small scales required for the development of parallel electric fields due to these waves can be produced by nonlinear effects, ionospheric feedback and phase mixing, giving rise to the variety of scales observed in the aurora. The strong density gradients above the ionosphere produce the ionospheric Alfvén resonator, which supports the fast oscillations necessary to provide for the broadband acceleration of auroral electrons. While the situations is not as clear at Jupiter, the new data coming from the Juno extended mission, which will lead to auroral zone crossings at lower altitudes in the northern auroral zone, should help clarify the most important mechanisms for auroral particle acceleration. Future missions, perhaps involving multiple spacecraft crossing auroral field lines at Earth, would be very useful in providing more detailed understanding of the dynamic processes that produce the aurora.

## Data Availability

This manuscript is a review primarily of theoretical work, and so no new physical data was produced in this work. Information regarding the types of numerical codes used in this work can by found in the Data Repository for the University of Minnesota (DRUM) at 10.13020/7vd6-at92.
